# 3-D EM exploration of the hepatic microarchitecture – lessons learned from large-volume *in situ* serial sectioning

**DOI:** 10.1038/srep36744

**Published:** 2016-11-11

**Authors:** Gerald John Shami, Delfine Cheng, Minh Huynh, Celien Vreuls, Eddie Wisse, Filip Braet

**Affiliations:** 1 School of Medical Sciences (Discipline of Anatomy and Histology) – The Bosch Institute, The University of Sydney, NSW 2006, Australia; 2 Australian Centre for Microscopy and Microanalysis (ACMM), The University of Sydney, NSW 2006, Australia; 3 Department of Pathology, Amphia Hospital, Breda, The Netherlands; 4 Maastricht Multimodal Molecular Imaging Institute, Division of Nanoscopy, University of Maastricht, 6200 MD Maastricht, The Netherlands; 5 Department of Internal Medicine, University of Maastricht, 6200, MD Maastricht, The Netherlands; 6 Cellular Imaging Facility, Charles Perkins Centre, The University of Sydney, NSW 2006, Australia

## Abstract

To-date serial block-face scanning electron microscopy (SBF-SEM) dominates as the premier technique for generating three-dimensional (3-D) data of resin-embedded biological samples at an unprecedented depth volume. Given the infancy of the technique, limited literature is currently available regarding the applicability of SBF-SEM for the ultrastructural investigation of tissues. Herein, we provide a comprehensive and rigorous appraisal of five different SBF-SEM sample preparation protocols for the large-volume exploration of the hepatic microarchitecture at an unparalleled X, Y and Z resolution. In so doing, we qualitatively and quantitatively validate the use of a comprehensive SBF-SEM sample preparation protocol, based on the application of heavy metal fixatives, stains and mordanting agents. Employing the best-tested SBF-SEM approach, enabled us to assess large-volume morphometric data on murine parenchymal cells, sinusoids and bile canaliculi. Finally, we integrated the validated SBF-SEM protocol with a correlative light and electron microscopy (CLEM) approach. The combination of confocal scanning laser microscopy and SBF-SEM provided a novel way to picture subcellular detail. We appreciate that this multidimensional approach will aid the subsequent research of liver tissue under relevant experimental and disease conditions.

The advent of electron microscopy (EM) modalities in the early 1930s{Knoll and Ruska^1^ #16} and their subsequent application throughout the biological sciences not long thereafter{#17^2^; Porter *et al*.^3^ #18; #17^2^; #17^2^; Ruska *et al*.^4^ #19}, heralded a new era in analysing the complex geometric arrangement of biological cells and tissues at the nanometre scale. Throughout the infancy of EM techniques, including both transmission (TEM) and scanning electron microscopy (SEM), the structural information gained was limited to two-dimensional (2-D) space, providing a mere snapshot of innately three-dimensional (3-D) structures.

Throughout the mid-1950s, advances in ultramicrotome design and specimen preparation procedures{Porter and Blum^5^ #25} facilitated the generation of 3-D ultrastructural information via the superimposition of consecutive 2-D TEM micrographs that were acquired from a serially sectioned specimen – a technique dubbed serial-section TEM (ssTEM){#387^6^; Gay and Anderson^7^ #6; Ware and Lopresti^8^ #9; Porter, 1953 #25}. Throughout the ensuing years, the application of ssTEM had a profound impact in generating 3-D data ranging from the whole organism to subcellular level{White *et al*.^9^ #24; #23^10^; #31^11^}. Currently, ssTEM is regarded as a partial effort in the generation of 3-D information, primarily due to the fact that it is a highly arduous and labour-intensive technique that is riddled with several sources of error, such as section compression and the loss of serial sections, which ultimately results in an incomplete data set. The tediousness of the technique is further compounded by the necessity to align image stacks in order to correct for misalignments that occur during the manual imaging of successive sections{Williams and Kallman^12^ #15}. Moreover, the generation of high-resolution axial resolution is fundamentally limited to the thickness of the resin section, and when considering the diminutive size of particular subcellular structures, it becomes apparent that serial sectioning between the conventional range (30–70 nm) may be insufficient in revealing the necessary ultrastructural information to fully encapsulate structures of interest.

More recently, automatic tape-collecting ultramicrotomy{Hayworth *et al*.^13^ #62} (commercially referred to as ATUM-SEM{Schalek *et al*.^14^ #61}) combined with backscattered electron imaging in a SEM has alleviated many of the difficulties associated with conventional ssTEM. The technique involves automated collection of ultrathin sections onto glow discharged Kapton tape, which are subsequently attached to silicon wafers prior to imaging. Integrated hardware and software solutions have further alleviated the need to manually image serial sections; allowing the automated relocation, imaging and 3-D reconstruction of regions of interest (ROIs). For a review of volume SEM imaging techniques, see {Titze and Genoud^15^ #60@@author-year}.

In the late 1960s, tilt-based electron tomography (ET) emerged as an alternative means to reconstruct the interior volume of biological structures, whereby specimens are incrementally tilted by virtue of a goniometer in a TEM through a range up to ± 70° and an image acquired at each 1–2° tilt increments{De Rosier and Klug^16^ #27; #59^17^; #28^18^}. The images are then aligned and back-projected in order to generate a 3-D reconstruction or tomogram of the specimen{Gan and Jensen^19^ #15}. The application of ET has been prolific in revealing high-resolution information of an array of diminutive structures including cells, organelles and macromolecular assemblies{Beck *et al*.^20^ #9; Lucic *et al*.^21^ #13; Shami *et al*.^22^ #12}. However, this approach is not suitable when sections thicker than 1–5 μm need to be reconstructed from a single acquisition{Baumeister *et al*.^23^ #30; Favard and Carasso^24^ #34}.

Contemporarily, serial block-face scanning electron microscopy (SBF-SEM) combined with automated ultramicrotomy within the chamber of a SEM has emerged as a technique that fills the gap between ssTEM and ET. SBF-SEM is a powerful technique facilitating the automated reconstruction of large volumes of resin-embedded biological material – up to a depth of 600 μm – with a lateral resolution matching the capabilities of a high-resolution field-emission SEM (FESEM). The first application of SBF-SEM originated with Leighton in 1981, who constructed a miniature ultramicrotome that functioned within the chamber of an SEM in order to generate successive images of the surface of an epon-embedded block of squid{#32^25^}. The technique was further refined by {Denk and Horstmann^26^ #26@@author-year} and subsequently commercialised by Gatan in a system referred to as the 3View. In the 3View, the surface of a block of resin embedded biological material is imaged by means of backscattered electron detection. Once an image has been acquired, the block is raised by as little as 15–200 nm to ensure focus is maintained, and an ultrathin section is cut from the surface of the block by virtue of an ultramicrotome equipped with a diamond knife within the specimen chamber of the SEM{Hughes *et al*.^27^ #35}. In this manner, it is possible to generate and assemble images of subsequent cut and re-cut surfaces of pre-aligned, large volumes of biological material.

To date, much of the literature employing SBF-SEM in biology has focused on the investigation of lipid-rich tissues such as the brain, which are contrasted using high concentrations and concomitant applications of heavy metal stains and mordanting agents prior to embedding{Denk and Horstmann^26^ #26; Wanner *et al*.^28^ #37; Jurrus *et al*.^29^ #39}. Limited data is currently available describing the harsh effects that such specimen preparation protocols could have on true ultrastructure. Therefore, we herein present a comprehensive appraisal of five sample preparation protocols applied to liver tissue, with a focus on determining the preservation quality and degree of contrasting of a range of subcellular structures, in order to determine the suitability of such protocols for the application of SBF-SEM. Next, we perform 3-D modelling and subsequent comparative morphometric analysis in order to holistically visualise and quantify key features of the hepatic microarchitecture, including parenchymal cells, the hepatic sinusoids and the bile canalicular network. Finally, we further demonstrate the applicability of the validated SBF-SEM approach for subsequent correlative light and electron microscopy studies, in order to combine the best practice of utilising selective molecular probes for confocal laser scanning microscopy studies (CLSM), with the 3-D ultrastructural resolving power of SBF-SEM.

## Results

### Appraisal of sample preparation protocols for SBF-SEM

**Figure 1:**
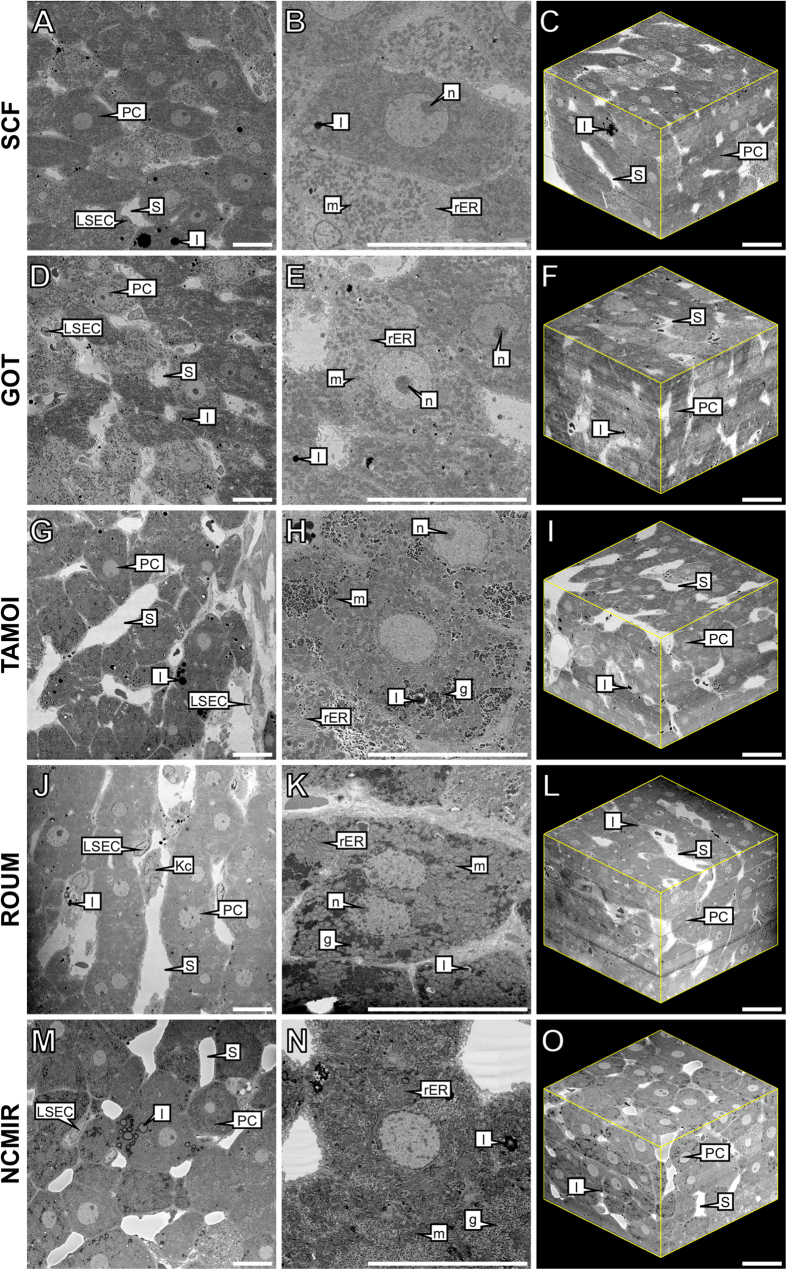
Comparative overview of the five experimental conditions assessed for their suitability for the application of SBF-SEM. Column 1 provides a global overview of rat liver tissue, revealing large polyhedral parenchymal cells (PC), separated by the hepatic sinusoids (S) that are lined by liver sinusoidal endothelial cells (LSEC). Kupffer cells (Kc) can also be visualised within the lumen of the sinusoids. Column 2 reveals notable variations in the presence, preservation quality and degree of contrasting of a range of subcellular structures, including mitochondria (m), rough endoplasmic reticulum (rER), lipid droplets (l), glycogen (g) and nucleoli (n). Column 3 corresponds to the 3-D reconstructed data of the five experimental conditions examined. Reconstructed 3-D volumes consist of 450 consecutive images (section thickness = 200 nm). Dimensions of 3-D volumes: *XY* = 122.61 μm *Z* = 90 μm. Total volume = 1,350,055.23 μm^3^. Scale bar = 20 μm.

During the first part of this study, we assessed the fixation quality and degree of contrasting of a variety of subcellular structures, based on the five sample preparation conditions tested. Such treatment appeared necessary to obtain sufficient contrast and to prevent charging artefacts for the large-volume reconstruction of the hepatic microarchitecture by means of SBF-SEM (Table 1, Fig. 1). It should be noted that backscattered electron micrographs are displayed with inverted contrast – an automated function during image acquisition – thus resembling the appearance of conventional TEM micrographs, thereby assisting with subsequent image analysis and interpretation.

**Figure 2:**
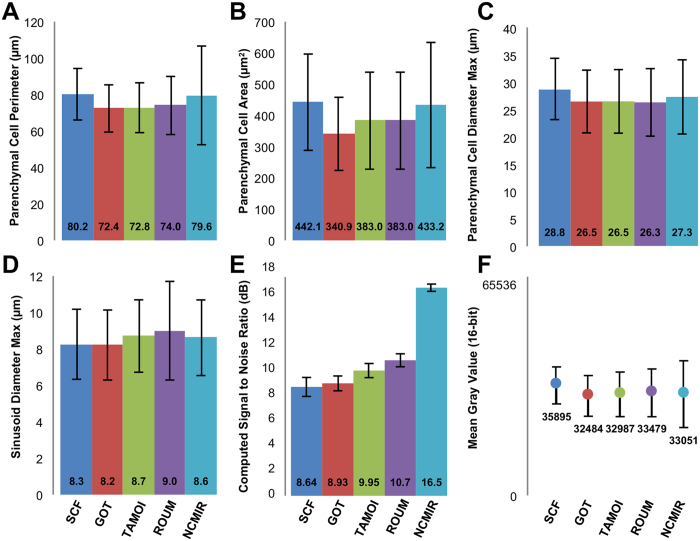
Summary of morphometric and quantitative data for entire parenchymal cells and hepatic sinusoids encapsulated within the acquired 3-D volumes. SCF, n = 148; GOT, n = 184, TAMOI, n = 171; ROUM, n = 172; NCMIR, n = 149.

At the gross cellular level, all samples displayed an analogous appearance, with parenchymal cells being characterised by a large, elongated polyhedral morphology, whilst sinusoids were patent and largely devoid of erythrocytes. A comparison of morphometric measurements on parenchymal cells and sinusoids, revealed only minor variations amongst the various protocols (Fig. 2).

At the ultrastructural level however, significant variations regarding preservation quality and the degree of contrasting of particular subcellular structures were evident amongst the five experimental conditions examined (Table 1).

Under the SCF and GOT protocols (Fig. 1A–F), tissues displayed good overall preservation quality and moderate contrasting of subcellular structures, including the plasma and nuclear membranes and lipid droplets. Of particular noteworthiness, was the absence of glycogen in the form of glycosomes or rosettes, throughout the parenchymal cell cytoplasm. Tissues prepared under the SCF and GOT protocols additionally revealed the lowest signal-to-noise ratio and global contrast measurement of the five experimental protocols examined (Fig. 2E,F).

Tissues prepared under the TAMOI protocol (Fig. 1G–I) revealed equal or superior fixation quality and contrasting of all subcellular structures relative to the SCF and GOT protocols. The most notable improvements in contrasting were observed amongst lipid-containing structures including cellular membranes, which were sharply delineated, in addition to large cytoplasmic lipid droplets, which appeared spherical in morphology and highly electron-dense. Relative to the SCF and GOT protocols, glycogen was visible throughout the cytoplasm of parenchymal cells, but displayed low overall staining.

Under the ROUM protocol (Fig. 1J–L), significant improvements were realised with regards to both preservation quality and the degree of contrasting of all selected subcellular structures relative to the three previous protocols. Of particular noteworthiness, was the greater level of contrasting of such structures as glycogen rosettes and the rough endoplasmic reticulum.

**Figure 3:**
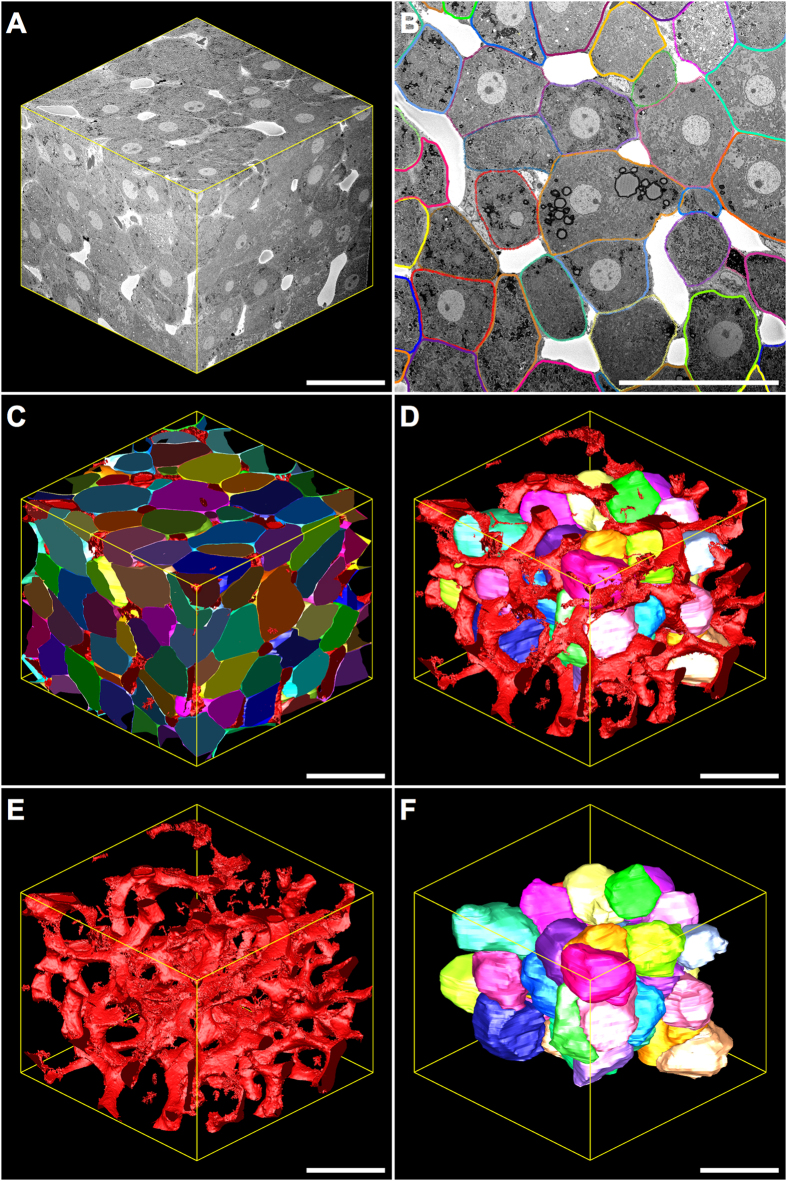
Three-dimensional reconstruction and corresponding model views of the hepatic microarchitecture prepared under the NCMIR protocol and imaged utilising SBF-SEM. (**A**) Reconstructed 3-D volume consisting of 500 consecutive images (section thickness = 200 nm). *XY* = 122.61 μm *Z* = 100 μm. Total volume = 1,503,394.77 μm^3^. (**B**) 2-D serial-section showing parenchymal cells (multi-coloured) arranged in cords, which have been segmented by means of manual tracing using IMOD. Modelled parenchymal cells (**C,D,F**) are formed via the superimposition of numerous segmented lines. Hepatic sinusoids (white spaces surrounding parenchymal cells) were segmented by means of isosurface thresholding. (**C**) Model view of partial and entire parenchymal cells (multi-coloured) and the hepatic sinusoids (red). (**D)** Model view of whole parenchymal cells (multi-coloured) surrounded by the tortuous hepatic sinusoids (red). (**E**) Model view of the hepatic sinusoids (red) within the reconstructed 3-D volume, which were segmented by means of isosurface/thresholding segmentation. (**F**) Model view of whole parenchymal cells (multi-coloured) within the reconstructed 3-D volume. Magnification = 796x Scale bar = 50 μm.

Tissues prepared under the NCMIR protocol (Fig. 1M–O) displayed the highest signal-to-noise ratio (1.9-fold improvement relative to the control) (Fig. 2E) and global image contrast (Fig. 2F), in addition to the highest preservation quality and contrasting of all subcellular structures of the five conditions tested (Table 1). Cellular membranes, including both the plasma and nuclear membranes were highly contrasted and clearly delineated from the cytoplasm. At high magnification, it was possible to observe such fine structural details as mitochondria cristae. The morphological appearance of lipid droplets was analogous to that of the four previous protocols, but revealed a denser staining periphery and a lighter staining core (Figs 1M and 3B). At all magnifications, images from tissues prepared under the NCMIR protocol showed little variation with regards to the maintenance of ultrastructural integrity, in comparison to the SCF and GOT protocols.

### *3*-D modelling and visualisation

**Figure 4:**
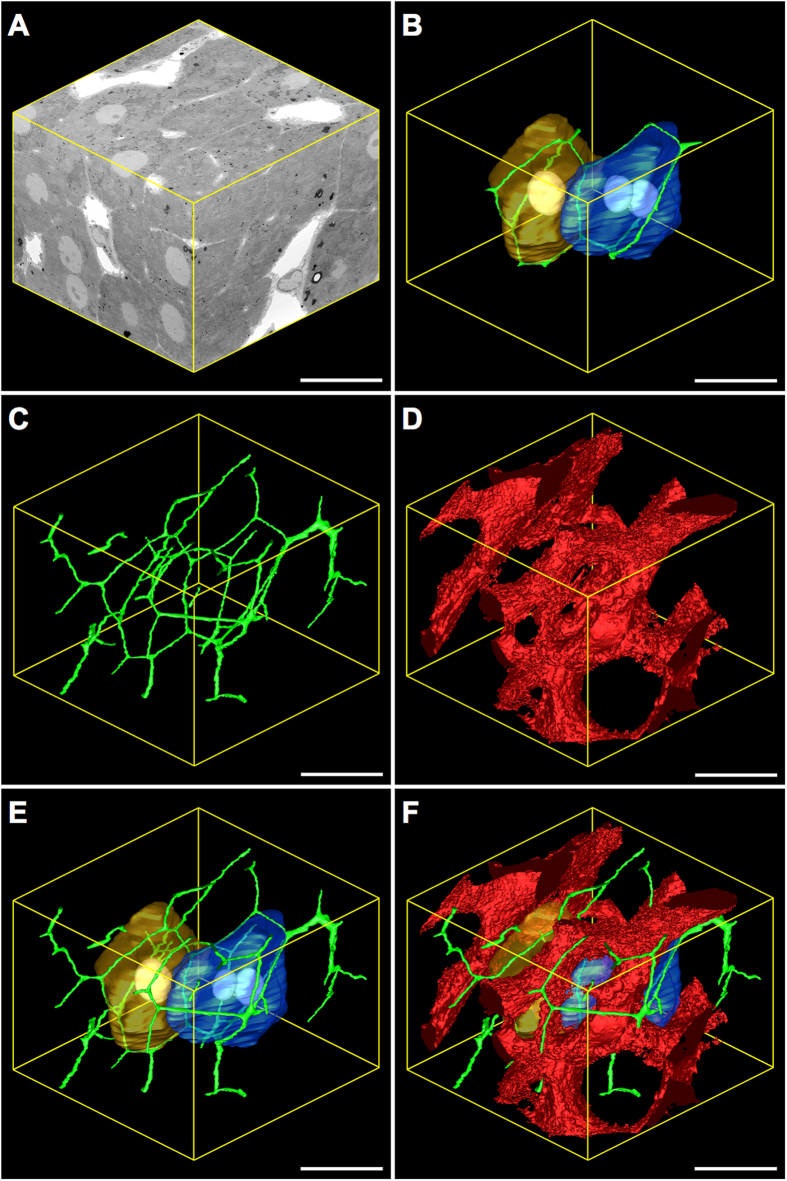
Three-dimensional reconstruction and corresponding model views of key tissue features of the hepatic microarchitecture. (**A**) Reconstructed 3-D volume consisting of 610 consecutive images (section thickness = 78 nm). *X* = 62.24 μm *Y* = 63.80 μm *Z* = 47.58 μm. Total volume = 188,935.99 μm^3^. Binned voxel size = 78 nm^3^. (**B**) Two adjacent parenchymal cells (gold (mononucleate) and (blue (binucleate)) surrounded by the bile canaliculi (green), which are formed via the apposing plasma membranes of bordering parenchymal cells. (**C**) Model view of the bile canalicular network (green). (**D**) Model view of the hepatic sinusoids (red). (**E**) Merged model view of (**B**,**C**). (**F**) Merged model view of (**B**–**D**). Magnification = 986x Scale bar = 20 μm.

Given the excellent preservation, contrasting and morphometric similarity to tissues prepared under conventional protocols, 3-D modelling was performed on tissues prepared under the NCMIR protocol (Figs 3 and 4).

**Figure 5:**
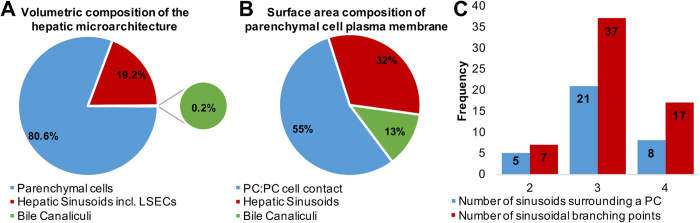
3-D morphometric data of rat liver tissue prepared under the NCMIR protocol. PC = parenchymal cell.

3-D modelling data revealed parenchymal cells as large, elongated polyhedral cells (Fig. 4B) that were radially arranged as plate-like sheets (Fig. 3C,F). They measured 27.3 ± 6.8 μm in diameter, had an average volume of 11,327.20 ± 2,968.60 μm^3^ and occupied 80.6% of the total liver microarchitecture by volume (Fig. 5A).

The mean surface area of the parenchymal cell plasma membrane measured 2,968.60 ± 457.15 μm^2^ and was divided into three distinct domains, of which 55% formed the parenchymal cell-to-parenchymal cell contact, 32% formed the parenchymal cell-to-sinusoid contact and 13% formed the parenchymal cell-to-bile canaliculi contact (Fig. 5B). At least two faces of an individual parenchymal cell were in contact with the hepatic sinusoids, with 14.7% of parenchymal cells facing two sinusoids, 61.8% facing three sinusoids and 23.5% facing four sinusoids (Fig. 5C).

The hepatic sinusoids, which are lined by a thin fenestrated endothelium, appeared as a network of tortuous channels that were largely devoid of red blood cells, as a consequence of jet-fixation (Figs 3E and 4D). They measured 8.81 ± 2.20 μm in diameter and occupied 19.2% of the liver microarchitecture by volume. 3-D analysis also revealed the frequency distribution of sinusoidal branching points from a given sinusoidal node – i.e. the connecting point where multiple sinusoidal vessels meet – with 11.7% of sinusoidal nodes involving two sinusoidal capillaries, 60.7% involving three sinusoidal capillaries and 27.9% involving four sinusoidal capillaries (Fig. 5C).

The bile canalicular system, appeared as a complex 3-D network formed by the apposing plasma membranes of adjacent parenchymal cells, of which a minimum of three facets of an idealised six-sided parenchymal cell were taking part in the formation of bile canaliculi (Figs 4B and 5B). In cross-section, the bile canaliculi appeared as empty circular profiles, measuring 1.02 ± 0.29 μm in diameter and occupied a total of 0.2% of the liver microarchitecture by volume.

### Correlative light and electron microscopy (CLEM)

In the second part of this study, we applied a CLEM approach – combining confocal laser scanning microscopy (CLSM), X-ray micro-Computed Tomography (micro-CT) and SBF-SEM – utilising the NCMIR protocol, previously determined to provide good preservation quality and contrasting of a range of subcellular structures (Table 1), relative to the five experimental conditions tested.

**Figure 6:**
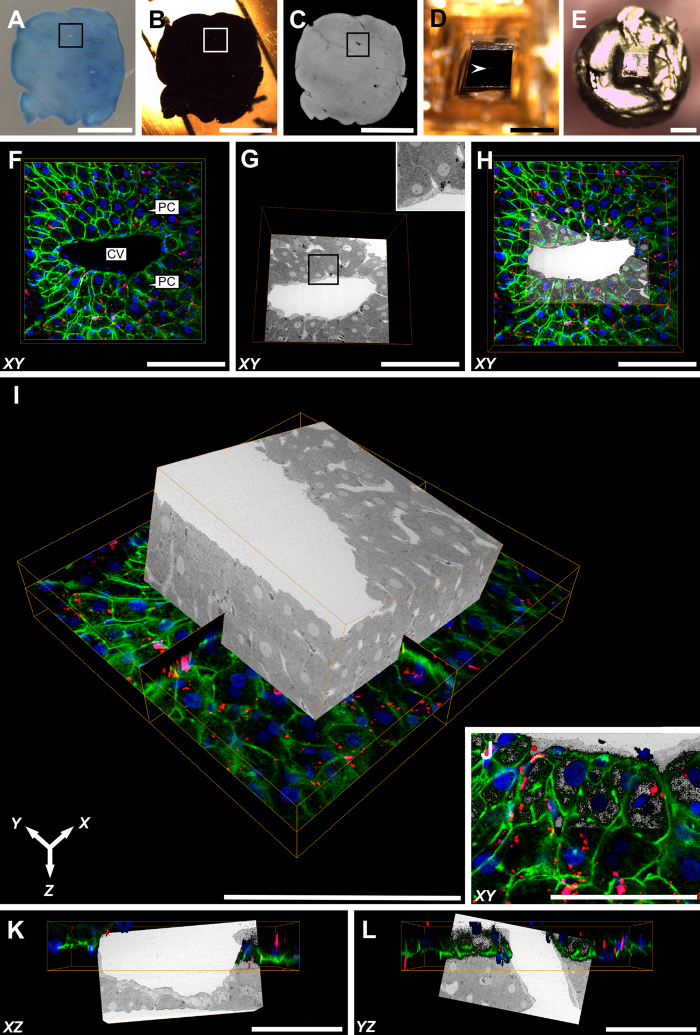
Three-dimensional CLEM: combining CLSM and SBF-SEM datasets. (**A**) Vibratome section of rat liver tissue proceeding CLSM imaging. The blue colour of the tissue is attributable to the histological staining properties of Nile Blue. Of particular note, Nile Blue produces red emission (see images F and H-L) when excited with 790 nm multiphoton excitation. The ROI (large vein) is indicated by a black bounding box. Conveniently, the scanned ROI appeared lighter in colour after CLSM imaging, relative to the surrounding tissue, assisting with relocation for subsequent correlation. (**B**) Epon-embedded sample prepared under the NCMIR protocol. The ROI corresponding to (A) is indicated by a white bounding box. (**C**) 3-D reconstructed x-ray micro-CT data, aiding in the confirmation of the ROI (black bounding box). (**D**) Precision trimmed epon block that has been centred, relative to the ROI (white arrowhead). (**E**) Gold coated sample, adhered to the specimen pin in preparation for SBF-SEM imaging. (**F**) *XY* confocal image showing a large central vein (CV) surrounded by plates of parenchymal cells (PC). The filamentous actin network predominantly underlying the plasma membrane is visible in green, nuclei appear blue and lipid droplets appear as red punctate or spherical structures distributed throughout the cell cytoplasm. (**G**) 2-D SBF-SEM image corresponding to the same area as shown in (**F**). A far more extensive array of structures are visible relative to (F), such as the hepatic sinusoids branching toward the central vein. (**G-inset**) Higher-magnification SBF-SEM image illustrating the high resolution capabilities of the SBF-SEM system. (**H**) Overlay of (**F**,**G**). (**I**) Overlayed 3-D representation of both CLSM and SBF-SEM volumes. (**J**) *XY* high-magnification overlay of CLSM and SBF-SEM datasets. (**K**,**L**) *XY* and *YZ* CLSM and SBF-SEM overlays. **Scale bars:** (**A**–**C**) = 3 mm; (**D**,**E**) = 700 μm; (**F**–**L**) = 100 μm.

After the incubation with Alexa Fluor^®^ 488 Phalloidin, Nile Blue and DAPI, CLSM revealed a filamentous actin network underlying the plasma membranes of both parenchymal cells and sinusoidal cells (green), the nuclei of mono- or binucleate parenchymal cells and mononucleate sinusoidal cells (blue) and intracellular lipid droplets (red) of varying size, most notably distributed throughout the parenchymal cell cytoplasm (Fig. 6F). The ROI – including a large central vein that was used as a morphological landmark for subsequent 3-D relocation – was noted due to its lighter-appearance upon prolonged exposure to the confocal and multiphoton lasers. In order to ensure the central vein of interest was not confounded with surrounding large-vessels, micro-CT was subsequently performed proceeding processing of the sample under the NCMIR protocol (Fig. 6C). Because of the non-destructive nature of micro-CT imaging, this allowed for the collection of 3-D data and subsequent virtual manipulation of the sample in order to confirm the ROI, prior to fine trimming of the epon block (Fig. 6D) for subsequent mounting (Fig. 6E) and SBF-SEM imaging.

The superimposition of both CLSM and SBF-SEM datasets by means of registration using Avizo, facilitated the correlation of fluorescently labelled structures, with their ultrastructural appearance, and association with adjacent subcellular structures; thus providing an unprecedented view of such structures across vast length scales in 3-D.

## Discussion

A fundamental distinction between SBF-SEM and conventional TEM is the necessity to utilise pre-embedding *en bloc* staining, in order to generate sufficient signal-to-noise and contrast for the visualisation of biological structures. Such a requirement is imposed by the limited penetrability of staining agents into the resin block, namely uranyl acetate and lead citrate. Moreover, due to the poor electrical conductivity of embedding media compatible with biological EM investigations, previous studies have emphasised the importance of utilising specimen preparation protocols that involve the successive application of heavy metal fixatives, stains and mordanting agents in order to prevent charging and mitigate beam damage{Deerinck *et al*.^30^ #5; Starborg *et al*.^31^ #4; Tapia *et al*.^32^ #41}. The issues of charging are particularly pronounced in cell nuclei, at high magnifications where the electron dose per unit area is increased, and when imaging “free resin spaces” such as extracellular spaces (e.g. blood vessels) due to the absence of conductive material. Recently, methods to improve sample conductivity have been reported via the inclusion of Ketjen black into the embedding media{Nguyen *et al*.^33^ #63}, or embedding samples in epoxy glue with silver particles proceeding infiltration with epon resin{Wanner *et al*.^34^ #64}. Such methods were revealed to primarily reduce charging in areas of low heavy metal content, and resulted in significant improvements in image quality. Further developments in commercially available electron-lucent conductive resins for biological samples, would assist in streamlining sample preparation for SBF-SEM; currently a rather laborious task, due to the need to employ protocols that involve multiple applications of heavy metal stains, in order to render samples conductive.

The relative infancy of SBF-SEM is illustrated by the limited literature currently available on a wide variety of different tissue types. From a range of sample preparation conditions, we selected and composed an appraisal of five experimental conditions for SBF-SEM. We demonstrated that the NCMIR protocol produced the most comprehensive results with regards to resistance to charging, stability under the electron beam, quality of preservation, degree of contrasting and visualisation of a range of subcellular structures, signal-to-noise ratio and global contrast (Table 1). Of note, was the appearance of lipid droplets, which revealed staining inhomogeneity (Figs 1M and 3B), opposing the typical appearance relative to conventional sample preparation methods (e.g. SCF){DiAugustine *et al*.^35^ #42; Angermuller and Fahimi^36^ #43}. Such an appearance is attributable to enhancing reactions such as ferrocyanide reduced osmium, combined with thiocarbohydrazide, which provides high contrast, however impedes secondary osmication{Hua *et al*.^37^ #65}. Such an effect ultimately results in a staining gradient or “coring effect”, whereby the periphery of lipid droplets stains more intensely, relative to the centre. The effect is particularly pronounced in large lipid droplets.

Samples prepared under the NCMIR protocol could also withstand higher accelerating voltages, facilitating an increased signal-to-noise ratio (Fig. 2E), greater distribution of grey values along the histogram (Fig. 2F) and an increased tolerance to charging and subsequently beam damage (Supplementary Figures S1 and S2). Ultimately, the combination of these factors permits imaging under higher magnifications, thereby improving lateral resolution via the generation of smaller pixel sizes.

In comparison, samples prepared under the SCF, GOT, TAMOI and ROUM protocols were far more susceptible to charging, as a consequence of poorer sample conductivity, thus requiring imaging under lower accelerating voltage and chamber pressure conditions (Table 2 and Supplementary Figures S1 and S2). Images of protocols containing low (SCF), intermediate (TAMOI) and high (NCMIR) amounts of contrasting agents acquired using fixed imaging conditions (accelerating voltage, 3.5 kV; chamber pressure, 28 Pa; objective aperture size, 30 μm; pixel dwell time, 12 μs) revealed the effects of such protocols on charging, beam damage and image contrast. This is further supported by a quantitative measurement of specimen current within with SEM, under the same sample imaging conditions previously mentioned; a higher current suggesting improved electrical grounding and thus reduced charging (Supplementary Figure S2). This data reveals a linear relationship (R^2^ value = 0.97455) between the amount of contrasting agents applied and their respective concentrations, of the various protocols examined, with respect to the aforementioned impact on sample charging and contrast generation; factors which are of critical importance for the acquisition of high-quality SBF-SEM data.

The suitability of the NCMIR method for the investigation of liver tissue is largely attributable to the vast array of staining, mordanting and contrasting agents employed, which facilitate the differential staining of various subcellular structures based on their biochemical composition (i.e. lipid, protein, carbohydrate etc.). Not only is this important for the selected visualisation of such structures, but also essential for the automated segmentation of structures based on differential greyscale values (e.g. modelling of the hepatic sinusoids by means of “isosurface” modelling). Of particular note, automated segmentation attempts on samples prepared under the SCF, GOT, TAMOI and ROUM methods (see also, vide infra), resulted in the capturing of structures of disinterest, particularly parenchymal cell nuclei, which displayed similar grey values to the hepatic sinusoids.

After having determined the superiority of the NCMIR method, we next performed segmentation and 3-D modelling of key tissue features of the hepatic microarchitecture. This allowed us to visualise the shape and 3-D arrangement of parenchymal cells, the hepatic sinusoids and the bile canalicular network both in isolation, and in combination with the various segmented structures at an unprecedented resolution (Figs 3 and 4). Segmented data facilitated the extrapolation of morphometric data regarding volumetric and surface area composition (Fig. 5). The validity of the 3-D morphometric measurements extrapolated from samples prepared under the NCMIR method, were firstly verified via a comparison of common morphometric parameters performed on all five sample preparation conditions examined (Fig. 2) and weighing our data against existing literature{Weibel *et al*.^38^ #56; Minnich *et al*.^39^ #55; Giuli *et al*.^40^ #54; Monteiro *et al*.^41^ #53; #52^42^}. Importantly, a significant advantage in acquiring morphometric SBF-SEM datasets, was the ability to fully encapsulate structures of interest, and thus obtain holistic morphometric data. Whilst such 3-D data has been previously achieved by means of confocal scanning laser microscopy{Hammad *et al*.^43^ #340}, the detail achieved by electron microscopy is far superior{Knott and Genoud^44^ #20}.

Despite the plethora of advantages offered by SBF-SEM with regards to the generation of large volumetric information in a relatively timely and automated manner, a major limitation of the experimental workflow was the segmentation of objects of interest. In our hands several segmentation approaches were evaluated, however it proved necessary to utilise a combination of automated and manual segmentation approaches in order to generate 3-D modelled and morphometric data of the parenchymal cells, hepatic sinusoids and bile canaliculi network. Segmentation of the hepatic sinusoids was performed by means of isosurface or thresholding segmentation, which classifies objects based upon a specific or a range (i.e. threshold) of pixel intensities. Given the relative homogenous pixel intensities of the sinusoids, in addition to the significant differential in grey values relative to the surrounding parenchyma, it was relatively easy and swift to delineate these structures (Figs 3E and 4D). Unfortunately, this approach was not suitable for the segmentation of parenchymal cells and the bile canaliculi (Fig. 3B,E,F). Accordingly, manual segmentation of these structures was performed, which involved computer assisted tracing of high contrast lines within 3dmod. The software then additively superimposes the numerous lines comprising an individual object, which are subsequently rendered for 3-D visualisation. When considering the significant size and number of image slices composing a single dataset, it becomes readily apparent that this is a labour intensive task, often spanning many weeks or months depending on the nature and aims of the investigation, and the level of detail required. Recently, increasingly automated interactive segmentation approaches such as “Microscopy Image Browser” (MIB){Belevich *et al*.^45^ #70} and “Ilastik”{Sommer *et al*.^46^ #71} have been developed, alleviating the need to perform manual segmentation in many investigations, and increasing the practical volume from which 3-D modelled and morphometric data can be generated.

Given that no single microscopy modality is capable of depicting biological organisms and their comprising structural constituents amongst the vast length scales throughout which they exist, it is becoming increasingly popular to utilise combined or correlative microscopy approaches in order to exploit the respective advantages of specific imaging modalities (e.g. light, electron, x-ray, scanning probe). By employing a correlative light an electron microscopy approach, we were able to harness the respective advantages of both light and electron microscopy modalities in order to generate complementary data not conferred by either technique if used in isolation.

CLSM offers the ability to selectively (e.g. fluorescent histological dyes) or specifically (e.g. molecular probes and immunofluorescence) stain structures of interest, non-destructively generate 3-D datasets of thick biological tissues, and do so in a relatively swift manner. Conversely, electron microscopy techniques, such as SBF-SEM, provide a much more detailed and complex depiction of cellular ultrastructure due to the broad staining properties of contrasting agents used in the specimen preparation of samples for EM, and the superior resolution achieved due to the significantly shorter wavelength of electrons relative to photons{#44^47^; #45^48^}. The strength of such an approach primarily lies in the generation of complementary and holistic 3-D structural information, which ultimately transcends conventional 2-D light or EM imaging approaches, in that it alleviates the potential for the misinterpretation of 3-D structures that may not be entirely encapsulated within a thin paraffin or resin section.

The ultimate significance of the CLEM approach presented herein, is its universal applicability to any targeted structure of interest – assuming the existence of an appropriate fluorescent probe. Consecutive processing of the sample under the proven NCMIR method would then allow for the generation of high-resolution 3-D EM data for subsequent correlation. The numerous heavy metal stains and mordanting agents inherent to the NCMIR method further makes this protocol an exceptional candidate for the preparation of samples for micro-CT, which generates contrast based on the differential X-ray density and scatting of biological structures. The use of micro-CT microscopy proved a powerful tool for the accurate relocation of the ROI – a central vein that acted as a morphological landmark – as a consequence of the sample being rendered opaque to light, proceeding sample preparation for EM. This practice further ensured that a greater volume of the sample could be collected in the SBF-SEM, due to difficulty in deciding where to capture data from the sample block face{Karreman *et al*.^49^ #69; Handschuh *et al*.^50^ #67; Bushong *et al*.^51^ #66}. Whilst micro-CT was simply used as a relocation tool, future studies could plausibly exploit the technique to further extract relevant structural information.

Alternative approaches to retain fluorescence within the block were attempted, however remain a topic of further investigation. Such attempts proved unsuccessful due to the requirement to omit high concentrations of osmium tetroxide which is known to quench fluorescence{Lucas *et al*.^52^ #57}, resulting in poor contrasting of subcellular structures and charging within the SEM. Additionally, the requirement to utilise acrylic resins such as LR White or HM20 are unideal for SBF-SEM, due to their tendency to crumble from the knife edge and fall back on to the block face, thus impeding imaging. Ultimately, we propose that the CLEM approach presented herein harnesses the advantages of generating high-yield fluorescence information from biological samples in an aqueous state, followed by the successive preparation for cross-correlative high-resolution SBF-SEM investigations (Fig. 6). Ultimately, such an approach could be applied in various healthy or diseased tissues in order to selectively localise specific events of interest (e.g. the detection of low-expressing markers at the onset of disease). Correlating such ROIs with SBF-SEM would subsequently provide an unprecedented view of such events at the highest resolution presently available, across vast length scales in multiple dimensions.

Herein, we provide a comprehensive appraisal of five different experimental conditions for the large-volume reconstruction of the murine hepatic microarchitecture by means of SBF-SEM (Fig. 1). Having deduced the most superior SBF-SEM sample preparation method (NCMIR) with regards to preservation quality, signal generation (Fig. 2) and contrasting of a range of subcellular structures (Table 1), we were able to characterise and quantify key features of the hepatic microarchitecture in 3-D including parenchymal cells, the hepatic sinusoids and bile canaliculi (Figs 3,4 and 5). Next, by integrating the validated NCMIR method for SBF-SEM with a CLEM approach (Fig. 6), we were able to selectively visualise subcellular structures including filamentous actin, cell nuclei and intracellular lipid droplets at an unprecedented resolution, length and volume scale in a novel manner. We propose the multidimensional CLEM approach presented herein can be universally applied to any relevant healthy or disease experimental model, in order to selectively elucidate specific structure-function relationships in 3-D.

## Materials and Methods

### Animals and primary fixation

For all protocols proceedingly listed, primary fixation of rat liver tissue was conducted in the following manner. All procedures were conducted in accordance with the guidelines and approval of the Animal Care and Ethics Committee of the University of Sydney.

Female Wistar rats (10–12 weeks of age) were housed in plastic cages at 21 °C with a 12-h light–dark cycle and were fed and watered *ad libitum*. Samples were fixed using a modified set-up of the “jet-fixation” method described by {Vreuls *et al*.^53^ #2@@author-year}. Liver biopsy samples were acquired using a Miltex^®^ biopsy punch equipped with a plunger (cat. no. T984-10, ProSciTech Pty. Ltd., QLD, Australia), providing tissue specimens of ~5 mm in length with a diameter of 1 mm. Biopsy samples were immediately transferred to physiological saline at 37 °C in a petri dish, wrapped in gauze and closed at one end with an artery clamp to facilitate handling of the sample. A primary fixative solution containing 1.5% glutaraldehyde (cat. no. C001, ProSciTech Pty. Ltd., QLD, Australia) in 0.067 M sodium cacodylate buffer (cat. no. 30118, BDH Chemicals Ltd., Poole, England), 1% sucrose (cat. no. 179949, Sigma-Aldrich, NSW, Australia) and 2 mM calcium chloride pH 7.4 at 37 °C was sprayed along the entire surface of the sample at a flow rate of 100 mL/min (total spraying time = 2 min) using a perfusion pump ending with an 18 G needle. The sample was rotated halfway during the procedure to ensure complete penetration of the fixative into the tissue. Following jet-fixation, the tissue was cut into 1 mm^3^ blocks, immersed in the primary glutaraldehyde fixative and allowed to react for a total of 1 h. Samples were subsequently rinsed with 0.1 M sodium cacodylate buffer containing 1% sucrose pH 7.4 (washing buffer) at room temperature (RT) (3 × 5 min) in preparation for subsequent processing.

### Fixation protocols

Proceedingly, the experimental details of the different tissue post-fixation and contrasting methods assessed in this study are described in-depth. Including the standard sample preparation for TEM/SEM/EM (i.e. control), tissue samples were prepared for SBF-SEM studies using five different sample preparation approaches:

#### Standard chemical fixation (SCF)

Samples were post-fixed with 1% aqueous osmium tetroxide (cat. no. C010, ProSciTech Pty. Ltd., QLD, Australia) in 0.1 M sodium cacodylate buffer containing 1% sucrose for 1 h at RT in darkness. Post-osmication, samples were rinsed with washing buffer (3 × 5 min) at RT, dehydrated, embedded and mounted as proceedingly outlined.

#### Glutaraldehyde–osmium tetroxide–tannic acid (GOT)

Samples were prepared as described under the “standard chemical fixation protocol”, however proceeding the osmication and rinsing steps, tissues were incubated in freshly made, filtered 1% tannic acid (cat. no. 2513/3, Polaron Equipment Ltd, Hertfordshire, England) in 0.1 M sodium cacodylate buffer containing 1% sucrose at RT for 1 h. Samples were rinsed with washing buffer (3 × 5 min) at RT, dehydrated, embedded and mounted as proceedingly outlined.

#### Tannic acid mediated osmium impregnation (TAMOI)

Samples were prepared using a modification of the TAMOI protocol outlined by {Jiménez *et al*.^54^ #3@@author-year} for the specific visualisation of plasma membrane associated specialisations. Tissues were post-fixed in 1% aqueous osmium tetroxide and 1.5% potassium ferrocyanide (cat.no. 74037, Univar, Australia) in 0.1 sodium cacodylate buffer containing 1% sucrose on ice in darkness for 1 h. Post-osmication, samples were rinsed with washing buffer (3 × 5 min) and incubated in freshly made filtered 1% tannic acid in 0.1 M sodium cacodylate buffer containing 1% sucrose at RT for 1 h. Samples were rinsed with ultrapure water (3 × 5 min) and post-fixed for a second time with 1% aqueous osmium tetroxide in ultrapure water on ice in darkness for 30 min. Proceeding secondary osmication, samples were rinsed with ultrapure water (3 × 5 min) at RT, dehydrated, embedded and mounted as proceedingly outlined.

#### Reduced osmium and en bloc uranyl acetate method (ROUM)

Samples were prepared using a modification of a protocol by {Starborg *et al*.^31^ #4@@author-year} to “determine collagen fibril size and 3-D organisation”. Tissues were post-fixed in 2% aqueous osmium tetroxide and 1.5% potassium ferrocyanide in 0.1 M sodium cacodylate buffer containing 1% sucrose for 1 h at RT. Samples were thoroughly rinsed in ultrapure water (3 × 5 min) and incubated in two changes of freshly made filtered 1% tannic acid in 0.1 M sodium cacodylate buffer containing 1% sucrose for a total of 4 h at 4 °C. Tissues were rinsed with ultrapure water (3 × 5 min) and secondarily post-fixed in 2% aqueous osmium tetroxide at RT for 40 min. Tissues were successively rinsed with ultrapure water (3 × 5 min) and incubated in 1% aqueous uranyl acetate (cat. no. C079, ProSciTech Pty. Ltd., QLD, Australia), at 4 °C in darkness overnight. Samples were rinsed with ultrapure water (3 × 5 min) at RT, dehydrated, embedded and mounted as proceedingly outlined.

#### National Centre for Microscopy and Imaging Research (NCMIR) protocol

Samples were prepared using a modification of the NCMIR method for “3-D EM for the preparation of biological specimens for serial-block face scanning electron microscopy (SBF-SEM)” {Deerinck *et al*.^30^ #5}. Samples were post-fixed in 2% aqueous osmium tetroxide and 1.5% potassium ferrocyanide in 0.1 M sodium cacodylate buffer containing 1% sucrose and 2 mM calcium chloride on ice in darkness for 1 h. Samples were subsequently washed with ultrapure water (3 × 5 min) and incubated in freshly prepared 1% aqueous thiocarbohydrazide (cat. no. C076, ProSciTech Pty. Ltd., QLD, Australia) for 20 min at RT. Samples were rinsed in ultrapure water (3 × 5 min) at RT and incubated in 2% aqueous osmium tetroxide in darkness for 30 min at RT. Tissues were successively rinsed with ultrapure water (3 × 5 min) and incubated in 1% aqueous uranyl acetate at 4 °C in darkness overnight. Samples were rinsed with ultrapure water (3 × 5 min) at RT and incubated in a freshly prepared solution of Walton’s lead aspartate containing 0.66% lead nitrate (cat. no. 280, Univar, Ajax Chemicals, NSW, Australia) in 0.40% aqueous L-aspartic acid (cat. no. A9256, Sigma-Aldrich, NSW, Australia) at 60 °C for 30 min. Samples were rinsed with ultrapure water (3 × 5 min) at RT, dehydrated, embedded and mounted as proceedingly outlined.

### Tissue dehydration, embedding and specimen mounting

Tissues were dehydrated in a graded series of ethanol concentrations including: 30% and 50% for 5 min and 70%, 90%, 100% in two changes for 10 min each at RT. Following dehydration, liver tissue was progressively infiltrated with one change of hard-grade epon:ethanol (25%, 50%, 75%) for 3 h. Tissues were placed in 100% epon overnight and then into fresh 100% epon for 3 h. Tissues were transferred to BEEM^®^ capsules in pure epon and polymerised at 60 °C for 48 h.

**Figure 7:**
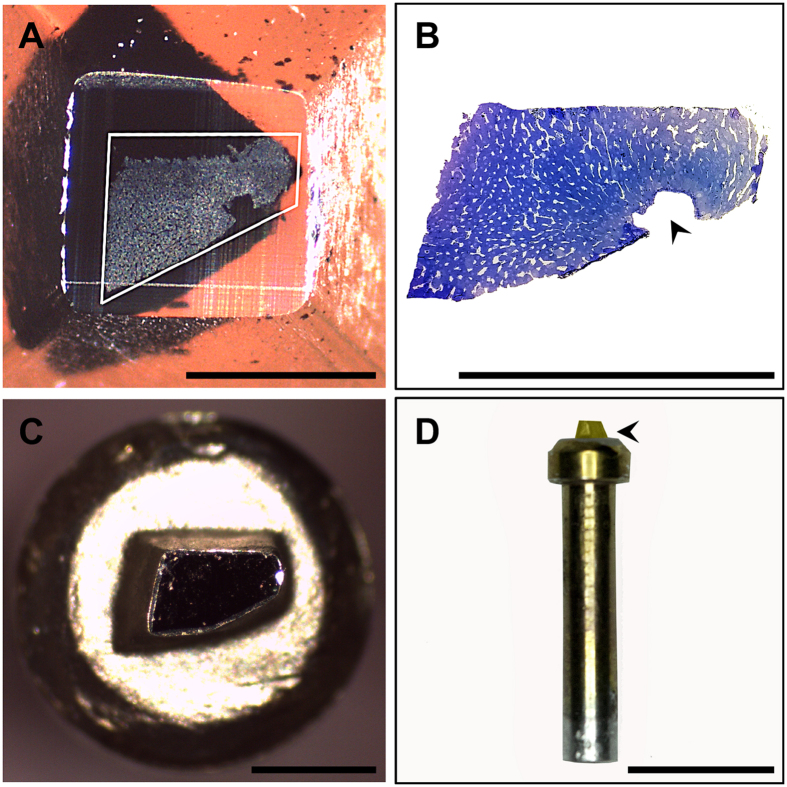
Stages of specimen mounting for SBF-SEM. (**A**) Exposed surface of resin-embedded rat liver tissue visible along the sectioned block face (white trapezoid). (**B**) Corresponding semithin section (0.5 µm-thick) showing a large vein (black arrowhead) surrounded by anastomosing sinusoids (white spaces). (**C**). The precision trimmed block has been centred and adhered to the specimen pin (top view) using cyanoacrylate glue. The exposed edges of the tissue were pained with silver paint, after which the entire block was gold coated in order to assist with alignment of the specimen within the SBF-SEM. (**D**) Longitudinal view of the specimen pin with gold-coated sample mounted atop (black arrowhead) in preparation for SBF-SEM imaging. Scale bar (**A**–**C**) = 500 μm, (**D**) = 5 mm.

Tissue blocks were sectioned with a Leica EM UC7 ultramicrotome (Leica, Heerbrugg, Switzerland) equipped with a glass knife to reveal the underlying tissue and trimmed with an injector blade to remove excess resin (Fig. 7A). Semithin survey sections (~0.5 μm thickness) were generated, stained with filtered 0.5% toluidine blue (cat no. C078, ProSciTech Pty. Ltd., QLD, Australia) in 1% aqueous sodium tetraborate (cat. no. 20716, Univar, Australia) and observed with a bright-field light microscope (40x, NA 0.65, Olympus BH-2) in order to verify complete exposure of the tissue along the block face and to determine adequate fixation quality.

Epon-embedded tissue blocks measuring approximately 1 mm^3^ were mounted onto aluminium specimen pins (Gatan, Pleasanton, CA) using cyanoacrylate glue (Turbobond Powerglue, Tewantin, QLD, Australia) and blocks were precision trimmed using an injector blade under a dissecting microscope, to ensure that tissue was exposed on all four lateral sides. In order to improve conductivity, the exposed edges of tissue blocks were painted with silver paint (cat. no. 16062, Ted Pella, Redding, CA, USA) and once dried, the entire specimen (block face and lateral sides) was sputter coated with a 30 nm thick layer of gold to assist with alignment and initial focusing within the SBF-SEM (Fig. 7C–D).

### Serial-block face scanning electron microscopy (SBF-SEM)

Backscattered electron images (8192 × 8192 pixels; *XY* pixel size 15 nm, *Z* pixel size (slice thickness) 200 nm, pixel dwell time 12 μs (i.e. the exposure time of an individual pixel to the electron beam)) were acquired using a variable pressure, field emission scanning electron microscope (Sigma VP, Carl Zeiss, Australia) at a fixed working distance of 4.3 mm. Imaging conditions were optimised in order to minimise charging and maximise brightness and contrast for each respective protocol (Table 2).

Once an ideal specimen preparation protocol had been determined, an additional SBF-SEM acquisition was performed using a thinner slice thickness (78 nm) for the purpose of high-resolution 3-D visualisation and morphometric analysis (Fig. 4).

### Correlative light and electron microscopy (CLEM)

For fluorescence microscopy, a strip of liver tissue measuring ~3 mm × 3 mm × 15 mm was fixed by means of jet-fixation at 37 °C, in a solution containing 4% formaldehyde (cat. no. C007, ProSciTech Pty. Ltd., QLD, Australia) and 0.4% glutaraldehyde in 0.067 M sodium cacodylate, 1% sucrose and 2 mM calcium chloride. Proceeding jet-fixation, liver tissue was cut into blocks measuring ~3 mm^3^ and allowed to react in the primary fixative for 3 h at RT. Tissue blocks were washed (3 × 5 min) with 0.1 M phosphate buffer solution (PBS) pH 7.4, mounted to the Vibratome^®^ specimen holder using cyanoacrylate glue, and subsequently immersed in a bath containing 0.1 M PBS at RT. Samples were sectioned by means of automated vibrating blade microtomy (Leica VT1200 S, Heerbrugg, Switzerland) using the following specifications: knife amplitude 2 mm/second, knife travel speed 1 mm/second, knife angle 9°, section thickness 70 μm. Sections were subsequently placed in a 96-well plate (cat. no. CLS3599, Corning Costar, Sigma-Aldrich, NSW, Australia) and stained with 5 units/mL Alexa Fluor^®^ 488 Phalloidin (cat. no. A12379, Invitrogen Life Technologies, NSW, Australia) for 1 h at RT in darkness. Tissues were rinsed with 0.1 M PBS (3 × 5 min) and incubated in a filtered saturated solution of Nile Blue A, sulphate (cat. no. C1291, ProSciTech Pty. Ltd., QLD, Australia) for 1 h at RT in darkness. Tissues were thoroughly rinsed in 0.1 M PBS and stained with 1 μg/mL DAPI (cat. no. D1306, Invitrogen Life Technologies, NSW, Australia) for 20 min at RT in darkness. Proceeding fluorescence staining, samples were rinsed with 0.1 M PBS (3 × 5 min), placed in an 8 well 1 μm-thick cover glass bottom slide (cat. no. 80827, Ibidi^®^, Martinsried, Germany) and filled with 0.1 M PBS in preparation for fluorescence imaging.

#### Confocal and multiphoton laser scanning microscopy

Fluorescence data stacks of sections measuring 3 mm × 3 mm × 70 μm were recorded (1024 × 1024 pixels; *XY* pixel size 223.7 nm; Z pixel size (step size/optical section thickness) 461.6 nm) using a Leica TCS SP2 spectral confocal and multiphoton system (Leica, Heerbrugg, Switzerland) equipped with a Plan 25 × 0.95 NA water immersion objective. Proceeding fluorescence imaging, samples were transferred to a 24-well plate (cat. no. CLS3526, Corning Costar, Sigma-Aldrich, NSW, Australia) and processed as previously detailed under the NCMIR protocol. Tissue sections were embedded in the lid of a size 00 BEEM^®^ capsule (cat. no. 130, Ted Pella, Redding, CA, USA) to ensure the flatness of the section was maintained during polymerisation. Particular care was taken to ensure the sample did not flip during processing, so as to maintain the correct orientation of the sample for subsequent EM imaging.

#### X-ray micro-Computed Tomography (micro-CT)

Micro-CT was performed in order to aid in the relocation of the ROI between light and electron microscopy imaging modalities, and to determine the depth of sectioning required to remove excess resin overlying the sample prior to automated sectioning in the SEM. Micro-CT datasets were acquired using a MicroXCT-400 (Carl Zeiss, Australia) equipped with a 4x objective operating at 150 kV source power, 10 W source current, over a 180° rotation angle. A total of 1201 images were acquired with an exposure time of 5 s. Images (972 × 995 × 974 pixels (752 × 826 × 207 cropped pixel dimensions); isotropic pixel size 46.4 μm^3^) were reconstructed using XMReconstructor (version 7.0.2817, Xradia Inc., Pleasanton, CA, USA).

*SBF-SEM:* Backscattered electron images (6144 × 6144 pixels; *XY* pixel size 30 nm, *Z* pixel size (slice thickness) 200 nm, pixel dwell time 12 μs) were acquired as previously outlined.

### Image processing, morphometric analysis and correlative data processing

SBF-SEM data was reconstructed using ImageJ (version 2.0.0-rc-39/1.50b), an open source image processing software package{Schindelin *et al*.^55^ #40}. Datasets were resampled to a final voxel size of 90 nm × 90 nm × 200 nm (Figs 1 and 3), 78 nm × 78 nm × 78 nm (Fig. 4) and 60 × 60 × 200 nm (Fig. 6). Contrast enhancement was performed by means of histogram normalisation and images were denoised using the “non-local means filter”. Signal-to-noise ratio measurements were calculated using a plugin for ImageJ{#72^56^} in order to objectively compare image quality (Fig. 2E).

For 3-D modelling, visualisation and morphometric analysis, datasets prepared under the NCMIR protocol were processed using IMOD (version 4.5.8), a suite of image processing, modelling and display programs used for 3-D reconstruction and segmentation of tomographic data and EM serial sections{Kremer *et al*.^57^ #22}. Cellular structures including parenchymal cells, the hepatic sinusoids and bile canaliculi were segmented by manual tracing of high-contrast lines or “isosurface” modelling within 3dmod, a graphical user interface application that is bundled with the IMOD software package.

Quantitative measurements of segmented structures were analysed in Microsoft excel, and statistical analysis was performed with the FT-test, and considered biologically significant at the 0.05 confidence level (two-tailed).

Registration of correlative light and electron microscopy datasets (Fig. 6) was performed using Avizo (version 9.0.1, FEI, Australia). Both datasets were visualised using the “Orthoslice” view, which produced three 2-D slices along the *XY*, *XZ* and *YZ* planes. The SBF-SEM dataset was then manually shifted so that it closely matched the CLSM dataset. In order to refine the correlation, automated registration was performed using the “aniso-scale” transformation, which allows for three translations, rotation and scaling of the SBF-SEM dataset, in order to correct for specimen preparation-induced morphological variations between the two datasets. Sample transparency was adjusted in order to visualise the correlating ROIs, which was confirmed using gross tissue features such as a large central vein, sinusoids and parenchymal cells, as well as nuclei and lipid droplets (Fig. 6J).

## Additional Information

**How to cite this article**: Shami, G. J. *et al*. 3-D EM exploration of the hepatic microarchitecture–lessons learned from large-volume *in situ* serial sectioning. *Sci. Rep*. **6**, 36744; doi: 10.1038/srep36744 (2016).

**Publisher’s note:** Springer Nature remains neutral with regard to jurisdictional claims in published maps and institutional affiliations.

## Supplementary Material

Supplementary Information

## Figures and Tables

**Table 1:** Qualitative appraisal of different specimen preparation protocols with effects on the degree of preservation quality (pres.) and contrasting (cont.) of selected subcellular structures visible at the acquired magnification.

Cellular structures	SCF		GOT		TAMOI		ROUM		NCMIR	
	Pres.	Cont.	Pres.	Cont.	Pres.	Cont.	Pres.	Cont.	Pres.	Cont.
Plasma membrane	+	+	+	+	++	++	++	++	+++	+++
Rough endoplasmic reticulum	+	−	+	−	+	+	+++	++	+++	+++
Mitochondria	±	−	±	−	+	+	++	++	+++	+++
Lipid droplets	++	+	+	++	+++	+++	+++	+++	+++	++
Glycogen	nv	nv	nv	nv	+	+	+++	++	+++	+++
Nuclear membrane	+	±	+	+	++	+	+++	++	+++	+++
Chromatin	++	±	++	±	+++	++	+++	+++	+++	+++
Nucleoli	++	+	+	+	++	+	+++	+	+++	+++

Criteria used in assessing fixation quality based on morphology: (nv) not visible; (−) poor; (±) satisfactory; (+) good; (++) very good; (+++) excellent.

SCF, standard chemical fixation; GOT, glutaraldehyde-osmium tetroxide-tannic acid; TAMOI, tannic acid mediated osmium impregnation; ROUM, reduced osmium and en bloc uranyl acetate method; NCMIR, National Centre for Microscopy and Imaging Research.

**Table 2:** Summary of optimised imaging conditions to minimise charging and maximise brightness and contrast for each respective specimen preparation protocol.

	SCF	GOT	TAMOI	ROUM	NCMIR
**Accelerating voltage (kV)**	1.4	1.5	1.5	1.4	3.5
	5	10	9	7	28
**Objective aperture size (μm)**	60	60	60	30	30
