# Supplementary material for: 3-D EM exploration of the hepatic microarchitecture – lessons learned from large-volume in situ serial sectioning
Source: Sci Rep. 2016 Nov 11;6:36744. doi: 10.1038/srep36744 (PMC5105151; doi:10.1038/srep36744)
Supplement: Supplementary Information [file srep36744-s1.pdf]

3-D EM exploration of the hepatic microarchitecture – lessons learned from large-volume *in situ* serial sectioning

Gerald John Shami, Delfine Cheng, Minh Huynh, Celien Vreuls, Eddie Wisse and Filip Braet

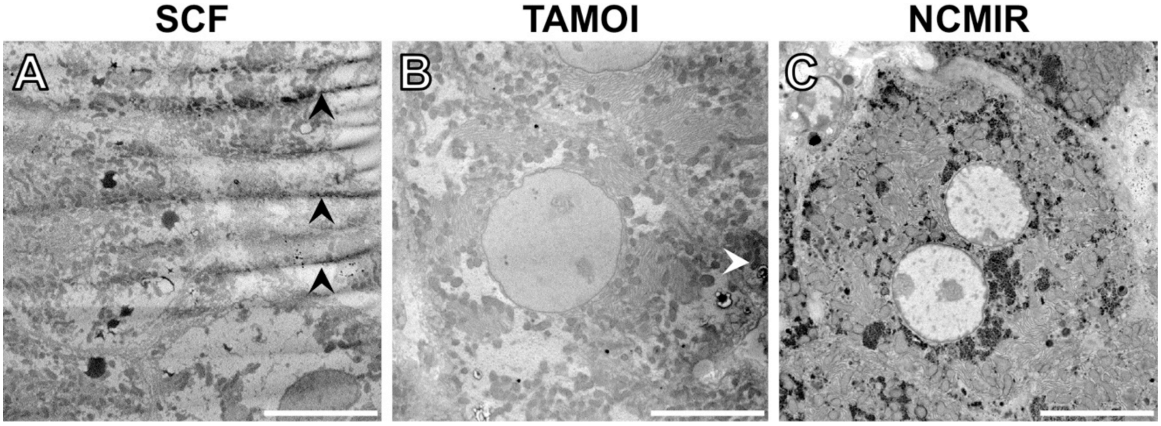

Supplementary Figure S1. Comparative overview of the effects of low (SCF), intermediate (TAMOI) and high (NCMIR) contrasting specimen preparation protocols in reducing charging and mitigating adverse electron beam interference. Imaging conditions were held constant for all three protocols (accelerating voltage, 3.5 kV; chamber pressure, 28 Pa; objective aperture size, 30 μm; pixel dwell time, 12 μs). **(A)** Under the SCF protocol the effects of charging and subsequently beam damage are evident along the block face, appearing as black lines that are particularly pronounced at the lateral sides of the image (black arrowheads). **(B)** Under the TAMOI protocol charging is reduced relative to (A), however is still apparent (white arrowhead). **(C)** Under the NCMIR intracellular charging is not evident. Scale bar = 10 μm.

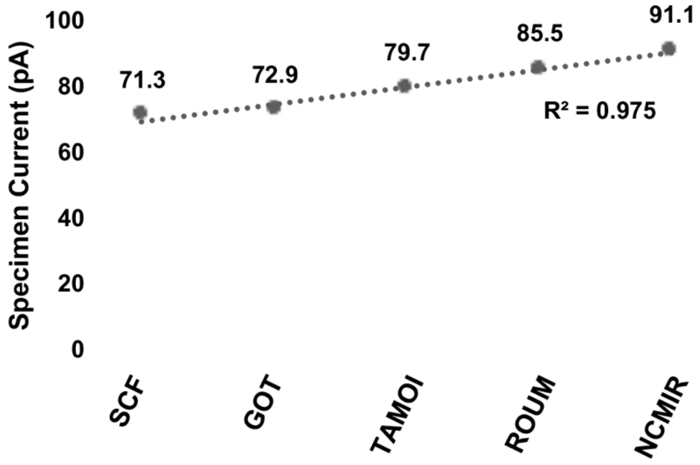

Supplementary Figure S2. Quantitative measurement of specimen current within the SEM, revealing the effects of the various protocols on electrical conductivity and subsequently charging. For each experimental condition, measurements (n=12) were acquired using the following imaging conditions (accelerating voltage, 3.5 kV; chamber pressure, 28 Pa; objective aperture size, 30 μm; pixel dwell time, 3 μs).
